# Supplementary material for: The Eosinophil-to-Lymphocyte Ratio Acts as an Indicator for Improvement of Clinical Signs and Itch by Upadacitinib Treatment in Atopic Dermatitis
Source: J Clin Med. 2023 Mar 12;12(6):2201. doi: 10.3390/jcm12062201 (PMC10058847; doi:10.3390/jcm12062201)
Supplement: Supplementary file 1 [file jcm-12-02201-s001.zip › jcm-2261299-supplementary.pdf]

Supplemental Table S1. The clinical indexes and laboratory parameters at week 0, 4 and 24 of upadacitinib treatment, and the comparisons between the stages (*n* = 65)

	Median [interquartile range]				*p*					
	Week 0	Week 4	Week 12	Week 24	Week 0 versus week 4	Week 0 versus week 12	Week 0 versus week 24	Week 4 versus week 12	Week 4 versus week 24	Week 12 versus week 24
EASI	20.4 [16.6-32]	4.4 [2.4-7.1]	3.85 [2.5-9.25]	4.2 [2.175-7.725]	0.0000000047**	0.000000005**	0.0000000091**	1	1	1
PP-NRS	8 [7-10]	2 [1-3]	2 [1-5]	2 [1-3]	0.00000002**	0.000000012**	0.000000127**	0.71	0.91	1
ELR	0.22 [0.13-0.47]	0.09 [0.06-0.09]	0.12 [0.07-0.22]	0.13 [0.07-0.3]	0.00000000059**	0.0000082**	0.002**	0.3924	0.0071	1
NLR	2.23 [1.65-2.81]	1.49 [1.09-2.14]	1.66 [1.4-2.29]	1.7 [1.31-2.37]	0.000013**	0.00082**	0.0177*	0.666	1	1
MLR	0.21 [0.17-0.28]	0.17 [0.12-0.22]	0.2 [0.14-0.26]	0.21 [0.17-0.29]	0.0000039**	0.1362	1	0.00158**	0.00044**	0.3588
PLR	156.6 [112.4-214]	139.8 [108.7-196.8]	170.871 [120.1-214.2]	152 [134.47-210.9]	0.0086**	1	1	0.0666	0.0852	1

*Statistically significant at $p < 0.05$, ** at $p < 0.01$, analyzed by Friedman's test.

EASI, eczema area and severity index; PP-NRS, peak pruritus-numerical rating score; ELR, eosinophil-to-lymphocyte ratio; NLR, neutrophil-to-lymphocyte ratio; MLR, monocyte-to-lymphocyte ratio; PLR, platelet-to-lymphocyte ratio
